# Traumatic Left Anterior Descending Artery Dissection in a Case of Pediatric Blunt Chest Trauma

**DOI:** 10.7759/cureus.31477

**Published:** 2022-11-14

**Authors:** Raymond I Okeke, Christian Saliba, Jasmin Cao, Felicia Lee, Bernard P Parrish, Jahnavi Nadella, Shin Miyata, Christopher Blewett

**Affiliations:** 1 General Surgery, Sisters of St. Mary (SSM) Health Saint Louis University Hospital, Saint Louis, USA; 2 Pediatric Surgery, Sisters of St. Mary (SSM) Health Cardinal Glennon Children's Hospital, Saint Louis, USA; 3 General Surgery, Saint Louis University School of Medicine, Saint Louis, USA

**Keywords:** emergency echocardiography, echocardiography, left anterior descending artery (lad), coronary artery dissection (cad), coronary dissection

## Abstract

Despite the reserve for recovery in pediatric trauma, blunt force chest trauma can cause insidious injuries that are easy to miss. Coronary artery dissection is a rare injury associated with blunt force chest trauma in the pediatric population and can present with vague or atypical symptoms. Pediatric patients can be unreliable in reporting symptoms, and providers can mistake coronary artery injuries for myocardial contusion, especially with improving laboratory tests and equivocal imaging. We report a case showing the importance of a high index of suspicion when presented with this trauma pattern in a pediatric patient.

## Introduction

Traumatic coronary artery dissections, although rare, can occur after blunt chest trauma in pediatric patients. Injury to the left anterior descending artery (LAD) is the most common, followed by the right coronary artery (RCA) [1]. Rapid acceleration or deceleration can cause a traumatic event [2]. Due to the severe potential complications and increased mortality associated with traumatic cardiac injuries, early detection is critical. However, early detection can be challenging due to the possible subtle or nonspecific presentation of clinical symptoms. In this case, we present a delayed diagnosis of LAD dissection in a pediatric patient following blunt chest trauma.

## Case presentation

A 13-year-old female with no significant past medical history presented to the emergency room with shortness of breath and hypoxemia. She had experienced blunt chest trauma from crashing into a buoy while riding in an inner tube attached to a boat. She was placed on a non-rebreather mask soon after arrival. X-ray and computed tomography (CT) imaging revealed bilateral lung contusions, pulmonary edema, a slight grade 3 liver laceration, a grade 2 spleen laceration, and trace pelvic hemoperitoneum. There was no evidence of injury to the sternum. An electrocardiogram (ECG) obtained for intermittent non-sustained ventricular tachycardia on telemetry showed a preliminary finding of ST segment elevations in V3-V5. Laboratory test results were notable for elevated troponin of 4.3 ng/mL that reached a peak of 139 ng/mL. An echocardiogram showed normal right ventricular (RV) systolic function and low normal left ventricular (LV) systolic function, as well as hypokinesis of the interventricular septum (IVS) and LV apex. We then performed a coronary CT angiogram (CCTA), which showed normal coronary arteries, mildly dilated LV with wall motion abnormalities, and moderately depressed global LV systolic function. Repeat troponins showed a downtrend. The patient’s chest pain resolved. She was on room air by day 3 of her hospital stay. We discharged her on a diagnosis of myocardial contusion for outpatient follow-up with cardiology. We managed abdominal injuries nonoperatively.

**Figure 1:**
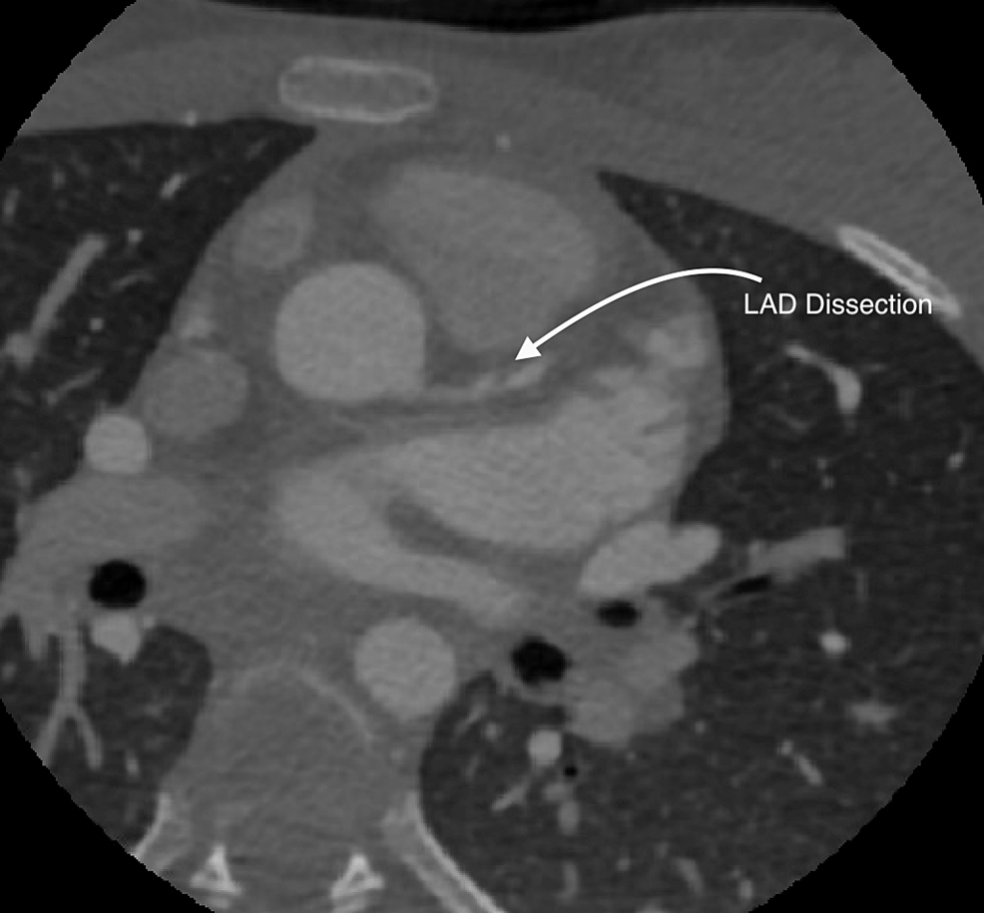
Cardiac CT angiogram showing focal narrowing of left anterior descending artery concerning for possible dissection flap CT: computed tomography

**Figure 2:**
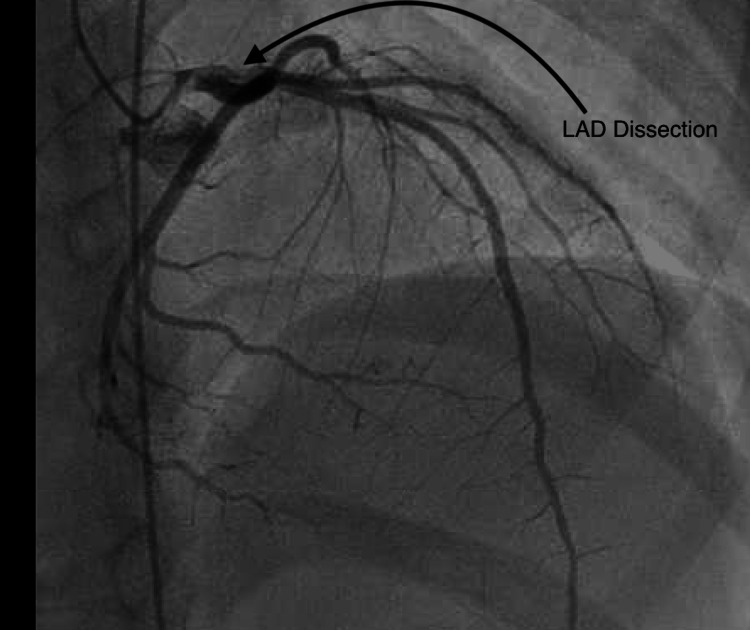
Left cardiac catheterization showing filling defect to proximal left anterior descending artery concerning for dissection

The patient was readmitted at a follow-up visit with cardiology five days later for shortness of breath and fatigue. An echocardiogram showed a left ventricular ejection fraction of 33%. Coronary CT angiogram (CCTA) showed focal narrowing of the proximal LAD (Figure 1) concerning for myocardial ischemia. We started the patient on lisinopril, Lasix, Aldactone, and metoprolol. She underwent a cardiac catheterization procedure (Figure 2) that showed proximal LAD dissection, nonviable anteroseptal myocardium, and anterolateral walls extending through the apex. The LAD dissection was concluded to have been from the time of her trauma, now 10 days old. A nuclear medicine stress test confirmed infarction of the LV free wall and apex. The patient was not considered a candidate for coronary artery bypass graft or recanalization because of the confirmed nonviable myocardium. Stenting was considered but not performed due to concerns for long-term patency. The patient was started on sacubitril/valsartan and aspirin and continued on Aldactone and metoprolol succinate. She was discharged with a wearable defibrillator in addition to these medications. She had no symptoms of chest pain or shortness of breath on discharge. She has had no acute coronary events since discharge and has no symptoms on follow-up clinic visits. Per the family’s request, other institutions were contacted for opinions on the patient’s case. The consensus is that percutaneous intervention or coronary artery bypass graft procedure will offer little utility as myocardium is nonviable. The placement of an implantable cardioverter-defibrillator in addition to current medications was recommended.

## Discussion

Coronary artery dissection as a complication of blunt chest trauma is highly uncommon, especially in pediatric patients. Most traumatic pediatric cardiac injuries include cardiac or pulmonary contusions, hemopneumothorax, and rib fractures [2], with coronary artery injuries making up only 2% of all cases [3]. However, despite its rare occurrence, it is crucial to recognize its possibility and any clinical findings that can lead to early detection and treatment due to the significant mortality and morbidity associated with this type of injury [4]. Failure to diagnose coronary artery injuries can lead to fatal complications such as myocardial infarctions, arrhythmias, or even sudden death [5]. Unfortunately, the presentation of coronary artery dissection often varies, with many patients presenting with asymptomatic, nonspecific, or delayed findings [1]. In our case, the patient complained of mild chest pain and left shoulder pain on her second hospital day. Subsequent workup revealed normal RV function and low normal LV function.

In general, the initial management of blunt cardiac injuries often involves screening tests such as chest X-ray and CT imaging, mainly used to identify any anatomical and vascular injuries. We recommend obtaining ECGs for all patients presenting with chest trauma as it provides a quick mechanism for evaluating primary cardiac function [6,7]. Cardiac markers such as troponins have solid prognostic value for assessing cardiac injury and risk of mortality [8]. We perform echocardiograms for patients with signs of myocardial injuries, such as elevated troponin levels, cardiac symptoms (chest pain or arrhythmias), or abnormal imaging [9]. If diagnostic studies and physical examination findings reveal concerns for cardiac vessel involvement, consider CCTA and coronary catheterization early, although the diagnosis may be rarer in pediatric trauma cases [10]. In pediatric trauma cases, further imaging tests are not mandated if the risk of coronary artery injury is ruled out [9,10]. However, elevated troponin and myocardial dysfunction may not be simply due to cardiac contusion. They should also raise clinical suspicion for other causes of cardiac injuries, such as coronary artery dissection or stenosis, despite the lack of obvious clinical symptoms such as chest pain, shortness of breath, or signs of hemodynamic instability [1]. The late presentation of myocardial ischemia in our patient could have been a sequela of an obstructing dissection flap. Coronary catheterization after the initial CCTA findings of LV wall motion abnormalities and depressed global LV systolic function may have identified the dissection at the index admission. However, with an improving clinical appearance and no evidence of the narrowing of coronary arteries on CCTA, such an invasive procedure may not be warranted at that time. We now recommend at least another 24 hours of monitored observation in such situations given the possibility of late myocardial ischemia with coronary artery dissections.

Unfortunately, pediatric patients’ treatment for coronary artery dissections is limited due to the low long-term success rates of coronary artery stenting and recanalization [4]. This is primarily due to the anatomical differences in children, such as narrower vessel diameters and increased arterial flexibility, which can increase complication rates of surgical intervention such as coronary rupture or thrombosis [1,10]. Thus, we recommend supportive treatment and close observation in many circumstances, especially in cases such as ours where the myocardium is nonviable.

## Conclusions

Despite the rare occurrence of coronary artery dissection as a complication of blunt chest injury in pediatric patients, its possibility cannot be overlooked. Diagnosis can be difficult due to the often vague or atypical clinical presentation. However, delayed diagnosis of coronary artery injuries may result in deleterious consequences, particularly in pediatric patients. Thus, we recommend rapid diagnostic testing, early intervention, and continued follow-up to prevent any complications of cardiac injury and improve the chances of recovery.
